# The Role of Growth Factors in Bioactive Coatings

**DOI:** 10.3390/pharmaceutics13071083

**Published:** 2021-07-15

**Authors:** Dragana Bjelić, Matjaž Finšgar

**Affiliations:** Faculty of Chemistry and Chemical Engineering, University of Maribor, Smetanova ulica 17, 2000 Maribor, Slovenia; dragana.bjelic@um.si

**Keywords:** bioactive coating, implant, bone tissue, controlled local release, growth factor, osseointegration

## Abstract

With increasing obesity and an ageing population, health complications are also on the rise, such as the need to replace a joint with an artificial one. In both humans and animals, the integration of the implant is crucial, and bioactive coatings play an important role in bone tissue engineering. Since bone tissue engineering is about designing an implant that maximally mimics natural bone and is accepted by the tissue, the search for optimal materials and therapeutic agents and their concentrations is increasing. The incorporation of growth factors (GFs) in a bioactive coating represents a novel approach in bone tissue engineering, in which osteoinduction is enhanced in order to create the optimal conditions for the bone healing process, which crucially affects implant fixation. For the application of GFs in coatings and their implementation in clinical practice, factors such as the choice of one or more GFs, their concentration, the coating material, the method of incorporation, and the implant material must be considered to achieve the desired controlled release. Therefore, the avoidance of revision surgery also depends on the success of the design of the most appropriate bioactive coating. This overview considers the integration of the most common GFs that have been investigated in in vitro and in vivo studies, as well as in human clinical trials, with the aim of applying them in bioactive coatings. An overview of the main therapeutic agents that can stimulate cells to express the GFs necessary for bone tissue development is also provided. The main objective is to present the advantages and disadvantages of the GFs that have shown promise for inclusion in bioactive coatings according to the results of numerous studies.

## 1. Introduction

As a body ages, the quality of the bone also deteriorates, which, along with other environmental factors and the state of health of the individual, influences the need to replace a joint with an artificial one [1]. Despite successful surgery and recovery, the problem of aseptic loosening and periprosthetic osteolysis may occur later, necessitating the replacement of the implant [2,3]. Within 10 years of the first artificial joint surgery, 4.4% of hip cases require artificial hip replacement, and 3.9% of knee cases require artificial knee replacement (i.e., revision surgery). Twenty years after the first surgery, artificial knee or hip replacement is required in 10.3% and 15.0% of individuals, respectively [4]. On average, replacement is required 15 years after the first surgery. Although some materials, including metals, polymers, and ceramics, are suitable for providing mechanical integrity, they are unsuitable for direct integration into host tissues, requiring approximately 0.9% of knee implants and 1.7% of hip implants to be replaced after one year [5,6,7].

The most common reasons for revisions include aseptic loosening, instability, infections, and osteolysis [8]. With appropriate implant design and modifications, it is possible to improve osseointegration and antibacterial activity, thus reducing the risk of infection [9,10,11]. For example, graphene oxide-modified titanium surfaces have been found to have antibacterial and osteogenic activity that increases with the number of graphene oxide layers [12]. TiO_2_ nanotubes have been found to inhibit bacterial adhesion without the use of antibiotics due to their topographical properties, while TiO_2_ also exhibited good corrosion resistance [13,14]. It has also been shown that TiO_2_ can act as an intermediate layer in the hydroxyapatite coating, which contributes to the strength of the coating [15]. The incorporation of Zn^2+^ and Ag^+^ into the coating also improved the corrosion resistance and osteogenic activity compared to the coating without these ions. Moreover, Zn^2+^ and Ag^+^ have also been found contribute to bacterial inhibition, as both are highly effective in killing bacteria in vitro and in vivo [16]. In addition, MgO nanoparticles have been reported to facilitate the prolonged release of Ag^+^, which (depending on Ag^+^ concentration) induced osteoblast differentiation and exhibited strong bactericidal capacity [17].

One of the main goals of early implant placement is to allow the bone to heal as quickly as possible. The ideal orthopaedic implant not only supports enhanced bone incorporation into the porous implant (osteoconduction) but also promotes rapid cell differentiation and growth (osteoinduction) [2,18,19]. Implant modifications, such as the addition of growth factors (GFs) to bioactive coatings, are required to prolong implant longevity and consequently reduce revision surgeries. Bioactive coatings enable the necessary osteoconduction and osteoinduction, thus extending the life of the orthopaedic implant [5,20]. The mere incorporation of GFs into the coating is not sufficient. The coating and implant must be designed in such a way that the GF is released in a controlled and prolonged manner [21,22]. Given that GFs are proteins, they have specific active binding domains that allow them to bind to various polymers and other therapeutic agents [23,24,25].

Understanding the composition of bone tissue and the mechanism of action of GFs is crucial for designing ideal coatings, as GFs regulate processes in the cell that are important for osteoconduction, osteoinduction, and osteogenesis. Among the most studied GFs is the group of bone morphogenic proteins (BMPs); more specifically, bone morphogenic protein-2 (BMP-2) [26,27,28]. BMPs are a group of GFs belonging to the transforming growth factor-β (TGF-β) superfamily, which regulate cell morphogenesis and proliferation in bone tissue. They induce increased expression of insulin-like GFs (IGFs), which also induce osteoblast proliferation and collagen type 1 expression, among other effects [29]. Although the osteoinductive activity of BMPs is stronger than that of TGF-β, the ability of TGF-β to regulate osteoblast growth and differentiation cannot be neglected. TGF-β stimulates the production of protease inhibitors or inhibits protease production, thereby affecting the formation of the extracellular matrix [30]. Angiogenesis is also important for healthy bone tissue. Basic fibroblast growth factor (bFGF) and vascular endothelial growth factor (VEGF) are strong inducers of angiogenesis, with the expression of the latter being induced by bFGF [31,32,33]. The wingless-type MMTV integration site family member 3A (Wnt3A) protein also belongs to the group of important GFs involved in osteogenesis. Together with BMP-2, Wnt3A induces the expression of osteoblastic differentiation marker genes [34,35,36]. In many clinical studies, recombinant human platelet-derived growth factor BB (rhPDGF-BB), in combination with allografts and alloplasts, has also been shown to be highly effective in healing periodontal defects [37].

The design of an implant with an optimal bioactive coating is still under development. Despite numerous in vitro and in vivo studies confirming the potential benefits of the desired effects of certain implants and coatings, many regulatory steps are required to translate them into clinical practice, resulting in high development costs [38]. Some of the most appropriate approaches to treat bone injuries are autografts and allografts, but they are limited by the volume of harvested bone and their purpose [39,40]. Among them, ceramics, stainless steel, titanium, and metal alloys are used as artificial joint replacements in clinical applications. There is a great need to improve their biocompatibility and osseointegration. Numerous in vitro and in vivo studies have reported on the use of growth factors. However, for implementation in clinical practice, it is imperative to develop an implant and coating that allows the controlled release of growth factors [41,42,43,44].

By incorporating bioactive coatings with various modifications, such as bone-inducing GFs, chemokines, inorganic nanoparticles (NPs), peptides, and other molecules, implants can be fabricated to be more compatible with bone tissue. However, the biogenic sources of bone tissue (autografts, allografts, and xenografts) should not be ignored, as they can be used to produce biomimetic thin films and are an excellent approximation of bone [45]. Bibliographic databases such as Scopus, Web of Science, and PubMed were used to obtain literature for this review. This review highlights the reported advantages of integrating GFs into bioactive coatings, including active compounds that induce the expression and synthesis of GFs within the cell. In addition, the disadvantages of the presence of GFs in combination with various coatings, as well as cell and tissue response, are also taken into consideration.

## 2. Bioactive Coatings and GFs

GFs are a key component in bioactive coatings for the maintenance of healthy bone texture and implant integration. GFs are proteins (soluble signaling molecules) that stimulate tissue growth and regeneration. They control cell response by specifically binding transmembrane receptors to targeted cells, which stimulates cell differentiation and proliferation in many species. Some GFs resemble hormones in that they can be secreted into the bloodstream, by means of which they are delivered to the target tissues. Although the production of hormones is limited to glandular tissue, GFs can also be produced by other types of tissue. GFs generally used in tissue engineering include bFGF, insulin-like growth factor-1 (IGF-1), transforming growth factor beta (TGF-β), VEGF, and various bone morphogenic proteins (BMPs). Some cytokines (small proteins secreted by one cell to regulate the function of another cell) may also function as GFs [46,47]. They represent a vital component in bioactive coatings as they are responsible for osteoconduction and osseointegration. Table 1 shows the advantages and disadvantages of each of the growth factors included in the coatings reported previously.

### 2.1. Bone Morphogenic Protein-2 (BMP-2)

BMP-2 is a well-known and efficient GF that binds to a specific cell membrane receptor and affects functions within the cell. The U.S. Food and Drug Administration (FDA) has approved recombinant human BMP-2, and it has been shown that it is an effective therapeutic agent in orthopedics [61,63,64,65].

BMP-2 has been incorporated into a plasma-polymerized ethyl acrylate coating known to trigger the spontaneous organization of fibronectin (FN) into nanonetworks. It enables high availability of the binding region for the transmembrane receptor integrin (FN III, 9–10 binding site) and the binding region for a GF (FN III, 12–14 binding site). Plasma-polymerized ethyl acrylate has been shown to be more suitable as a coating compared to polymerized ethyl acrylate applied by the spin coating technique, as BMP-2 is better absorbed into the FN network and thus strongly induces mesenchymal stem cell (MSC) differentiation [55]. Compared to the plasma-polymerized film coating on 3D-printed titanium implants, ion-assisted plasma polymer (IAPP) film coatings show greater bone inductive activity, which is a consequence of the higher density and stability of the reactive IAPP layer. An IAPP layer allows better adhesion of biomolecules, such as GFs, without hindering their bioactivity, which can happen during a reaction with reactive free radicals [54]. BMP-2 is an effective GF, but its weakness is its short half-life, i.e., 1–4 h [19]. This means that 1–2 mg per scaffold would be required for satisfactory efficacy, which far exceeds the physiological concentration of BMP-2, which is up to six orders of magnitude lower (ng/scaffold) [49,51,66]. The toxic concentration of BMP-2 that induces apoptosis depends on the cell type, but in the specific case of osteoblasts, it depends on the maturation state. It has been shown that a concentration of 200 ng/mL increases markers associated with apoptosis in MG63 cells, while it significantly induces apoptosis in the NHOst cell line of osteoblasts [60]. Thus, covalent immobilization by IAPP better retains the aforementioned GF on the implant surface compared to physically adsorbed BMP-2 [54]. Bone mesenchymal stem cells (BMSCs) showed enhanced proliferation and osteogenesis in the cultivation of BMP-2-loaded silk-coated hydroxyapatite nanocarriers [48].

The persistence of GFs and their controlled release are some of the major challenges facing medicine and bioscience. One possible solution is the use of porous biphasic calcium phosphate, consisting of hydroxyapatite and β-tricalcium phosphate in a ratio of 6:4, coated with collagen type 1. The pores are created by the addition of camphene (C_10_H_16_), and collagen type 1 serves to improve the cross-linking, i.e., the loading efficiency of BMP-2 and its controlled release. Collagen supplementation was found to reduce the initial release of BMP-2 from 85% to 55% in 24 h and to provide almost twice the carrying capacity [56]. Long-term release of BMP-2 can be achieved by the layer-by-layer coating technique with alternating polyanionic and polycationic layers and a final layer of type 1 collagen, into which BMP-2 is then incubated. The polyanionic layer consists of glycosaminoglycans such as heparin and chondroitin sulfate, and the polycationic layer is represented by chitosan [142]. A sufficient amount of BMP-2 can be achieved by incorporating chitosan hydroxypropyltrimethyl ammonium chloride into bioactive mineralized collagen coatings, loading more BMP-2 with a more controlled release due to electrostatic interactions (25% within 14 days) [57]. Chitosan coating can also be modified with carboxymethylation, which provides -COOH groups on the coating’s surface, which increases the antibacterial activity and enables conjugation with BMP-2 [58]. Chitosan beads of a size between 106 and 150 μm, filled with BMP-2, have also been shown to be effective as a component of a poly(lactic-co-glycolic acid) (PLGA) coating, as BMP-2 was slowly released from beads with pore sizes between 10 and 20 nm for 14 days in an in vitro experiment. An in vivo study with rats confirmed the efficacy of chitosan beads, as 26% more new bone tissue was formed in 6 weeks compared to empty chitosan beads [52]. Among other factors, the ability of GFs to induce cell attachment, growth, and osteodifferentiation is also important. BMP-2 and arginine-glycine-aspartate (RGD) peptides were identified as key components in the polydopamine-coated hydroxyapatite and PLGA composite. In vitro and in vivo studies have shown that the combination of RGD and BMP-2 significantly promotes cell adhesion and osteogenic differentiation [143].

**Figure 1:**
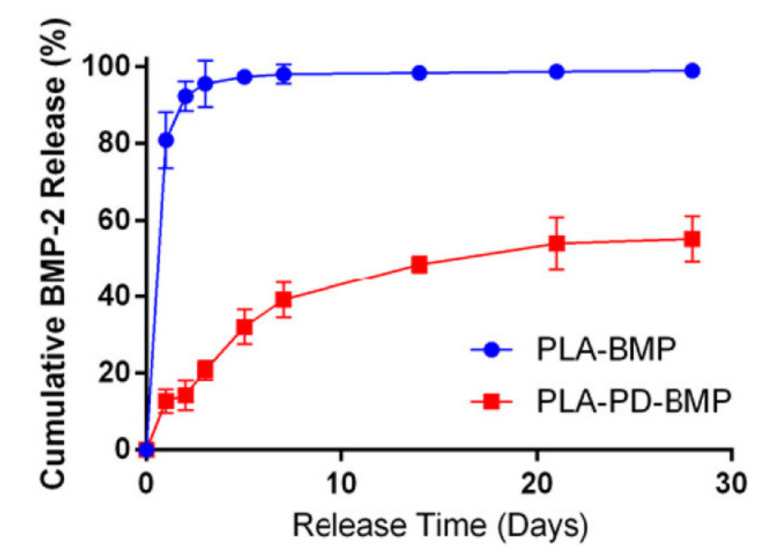
Release profiles of BMP-2 from PLA coating (PLA-BMP) and PLA coating with the addition of polydopamine (PLA-PD-BMP). Reprinted (adapted) with permission from [53], American Chemical Society, 2018.

Osteoblast differentiation triggered by BMP-2 can be accelerated by an appropriate dose of dexamethasone. In order to enhance this synergistic osteoinductive effect, small molecules of dexamethasone were encapsulated in silicate mesopores, and high molar mass BMP-2 was incorporated into the chitosan coating [50]. Polydopamine has been shown to be a very useful immobilizer for BMP-2 on a polylactic acid (PLA) substrate both in vitro and in vivo. The substrate was produced by 3D printing, which allows better mimicry of tissue composition. The presence of a polydopamine immobilizer was found to slow the release of BMP-2, of which approximately 55% was released within 28 days (Figure 1) [53]. The consistency and adhesion of the coating on the scaffold surface are also important, as they largely determine the mechanism of drug release and the rate of release from the polymer system. The encapsulation of BMP-2 in sodium carboxymethyl cellulose or hydroxypropyl methylcellulose coatings in combination with a poly(d,l-lactide) (PDLLA)/poly(ε-caprolactone) (PCL)/β-tricalcium phosphate scaffold can reduce such undesired losses [59]. BMP-2 GF can be incorporated into orthopaedic implants in two ways. In a comparative in vivo study on rabbits, BMP-2 was incorporated into PLGA and tricalcium phosphate implants using both an inclusive approach and a coating approach. In the case of the inclusive approach, the GF was incorporated into the scaffold during fabrication and, in the case of the coating approach, the fabricated scaffold was immersed in a prepared solution containing the GF. It was shown that bone regeneration was 72% higher in the inclusive approach [62].

**Figure 2:**
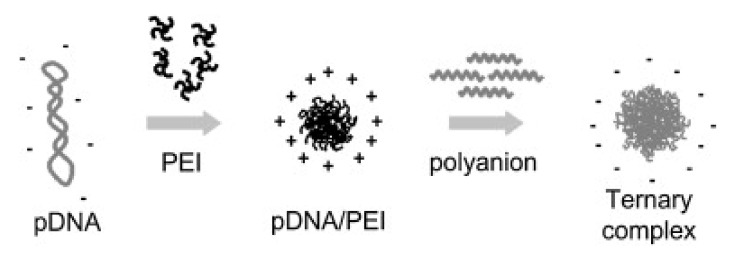
Schematic representation of the formation of the ternary complex. The components are pDNA, polyethyleneimine (PEI), and a polyanion. Reprinted with permission from [146], Elsevier, 2009.

On the other hand, the BMP-2 concentration should be sufficient, i.e., effectiveness requires high dosages. The latter, however, increases the risk of severe systemic toxic effects. Local delivery of BMP-2 is provided by the incorporation of copolymer-protected gene vectors (COPROGs) consisting of a plasmid deoxyribonucleic acid (pDNA) core electrostatically surrounded, through polycation (branched polyethyleneimine), by an anionic copolymer (PEG, Figure 2). The sequence required for BMP-2 protein synthesis is contained in pDNA. [144]. The toxicity of the polyethyleneimine particles [145] and the high concentrations of pDNA, which are unacceptable due to their cytotoxic, oncogenic, and mutagenic effects, are problematic [67,146,147].

### 2.2. Bone Morphogenic Protein-7 (BMP-7)

Bone morphogenic protein-7 (BMP-7), also known as osteogenic protein-1, is a GF of the TGF-β superfamily. It has been shown that more than 80% of BMP-7 can bind to titanium based scaffolds (99.99% purity), poly(ethyl acrylate)-coated titanium-based scaffolds, or poly(ethyl acrylate)-coated titanium-based scaffolds with the addition of FN. The addition of FN significantly affects osteogenic differentiation at low BMP-7 concentrations, which did not occur in the absence of FN at a BMP-7 concentration of 5 ng/cm2 [77]. In order to achieve a biological effect, such as osteogenic differentiation, ALP activity, osteocalcin production, collagen deposition, and extracellular matrix mineralization, a BMP-7 concentration approximately 10 times higher is required in the absence of FN [71,75]. The biological effect of BMP-7 depends on the type of cells exposed to it. In the case of adipose tissue-derived mesenchymal stem cells, BMP-7 can stimulate a chondrogenic phenotype by inhibiting the gene expression of runt-related transcription factor 2 (Runx2), osteopontin, and biglycan. Compared to BMP-7, BMP-2 has the ability to increase the expression of the above-mentioned genes and therefore stimulates osteogenic differentiation [72,76]. On the other hand, osteogenic differentiation can be achieved in pluripotent mesenchyme-derived C2C12 cells, where BMP-7 induces increased Runx2 expression [70]. An in vivo study using isolated human mesenchymal stem cells applied to β-tricalcium phosphate scaffolds and implanted in mice has also shown a difference in the efficacy of BMP-7 according to cell type. In the presence of low concentrations of BMP-7, mesenchymal stem cells isolated from reaming debris were found to have a greater osteogenic potential than those isolated from iliac crest bone marrow [81].

**Figure 3:**
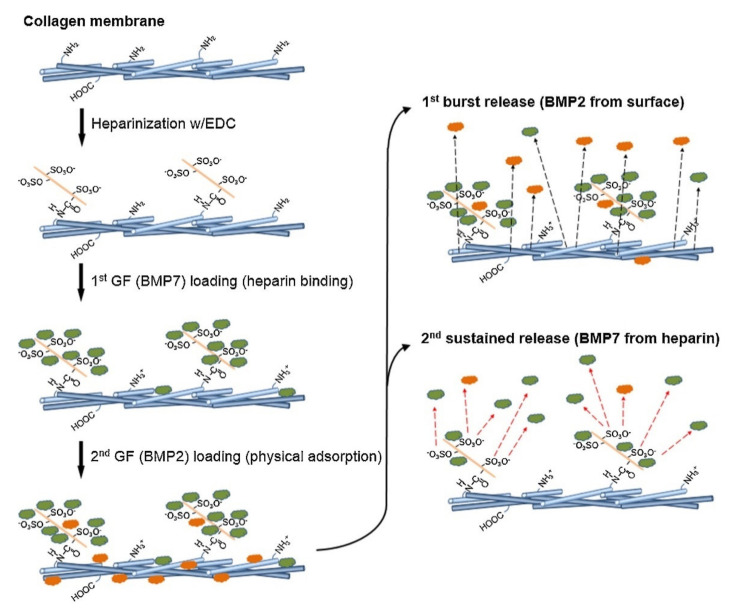
The process of heparinization, the loading of BMP-7 and BMP-2, and release. Reprinted with permission from [149], Elsevier, 2015.

In another in vitro study, the influence of different bone morphogenic proteins on the differentiation of bone marrow-derived human mesenchymal stem cells was compared, i.e., bone morphogenic protein-4 (BMP-4), bone morphogenic protein-6 (BMP-6), and BMP-7, with the cells grown on poly(propylene fumarate) scaffolds. The advantage of BMP-7 over BMP-4 and BMP-6 was shown in the mineralization, as it was significantly higher than in BMP-4 and BMP-6 regardless of the concentration [69]. In addition to BMP-2, the FDA also approved BMP-7 as a safe drug for clinical trials in 2004. Five years later, however, there was an about-face when the FDA decided not to recommend approval of the Stryker Corporation’s OP-1 Putty, a product that aids bone fusion in spine surgery [82,148]. The effectiveness of BMP-2, BMP-6, and BMP-7 has been compared and it has been reported that a BMP-2 surface concentration of 2.03 µg/cm^2^ was required for an osteoinductive effect, whereas BMP-7 achieved the same effect at just 1.06 µg/cm^2^. It was found that BMP-6 provided the best results among the BMPs, achieving the same efficacy at a surface concentration of 0.56 µg/cm^2^. Furthermore, the osteogenic activity of BMP-7 is annihilated when a complex of FN and BMP-7 is adsorbed onto titanium-hydroxyapatite surfaces compared to surfaces adsorbed with BMP-7 alone [73]. The addition of heparin to a collagen membrane was shown to provide the loading of a higher amount of BMP-7 and enable the desired controlled release. With the addition of BMP-7 to the heparinized membrane, a 60% binding efficiency was achieved. A binding efficiency of 40% was reported by the further addition of BMP-2 to this membrane. The cause of this amount of binding is the occupancy of the heparin-binding sites with BMP-7, which disables the effective binding of BMP-2 (Figure 3) and consequently results in a burst release of 75% of BMP-2 within 1 h. On the other hand, BMP-7 was slowly released over 28 days [149].

Just as osteoblasts represent the desired type of cells, fibroblasts are an undesirable type of cells that prevent the integration of the implant with bone tissue. BMP-7 has been shown to be a fibroblast inhibitor that reduces the proliferation and adhesion of fibroblasts and reduces the expression of fibrotic markers [74]. While it prevents the formation of fibroblasts, BMP-7 still promotes the formation of osteoblasts and the healing of cartilage tissue on porous tantalum scaffold [80]. The defect healing, mineral content, and mechanical strength of the bone tissue can be improved by, in addition to the presence of BMP-7 in the implant coating, combined treatment with subcutaneously injected bisphosphonates such as zoledronate [79]. An in vivo study on mice showed that BMP-7 improves the healing and quality of bone tissue in the case of osteoporosis, as it stimulates osteogenesis [68]. The importance of local delivery and long-term release is also reflected in the influence of BMP-7 on the healing rate of fractured sites. One of the most important findings is the bridging fusion mass, which has been observed at the site of the bone fracture, whereas there was no bridging fusion mass in the control group [78]. The formation of bone fusion mass, which consists, inter alia, of collagen and osteoblasts, can be induced by peptides that are shorter segments of the amino acid sequence of the entire BMP-7 [150].

### 2.3. Basic Fibroblast Growth Factor (bFGF)

**Figure 4:**
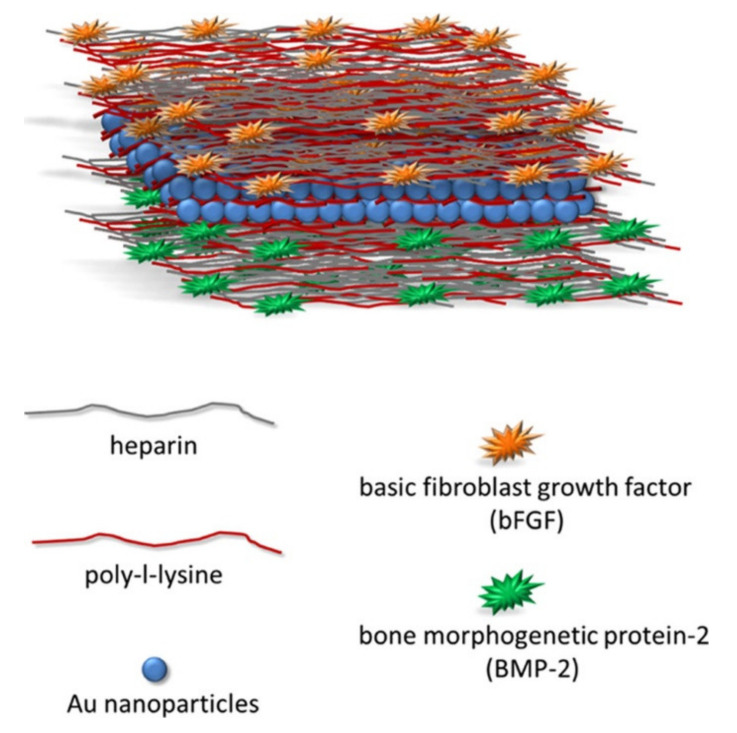
A schematic presentation of the components and structure of the multilayer coating described in [93]. Reprinted with permission from [93], American Chemical Society, 2017. https://pubs.acs.org/doi/full/10.1021/acsomega.6b00420 (accessed on 3 December 2020).

Prolonged release of GFs can be achieved by depositing gold NPs between layers in the bioactive coating. The highest ALP activity and increased expression of collagen type 1 was found in cells where PLA substrate was employed on which the bioactive coating was applied. This bioactive coating consisted of heparin/poly-L-lysine bilayers into which BMP-2 was immobilized (the lowest position), followed by six bilayers of poly-L-lysine/gold NPs (middle position) and six bilayers of poly-L-lysine/heparin immobilized by bFGF (in the topmost position, Figure 4). With such a composition and a layer-by-layer application technique, GFs were released continuously for 21 days, and low cytotoxicity was observed [93].

bFGF, also known as fibroblast growth factor-2, is mainly expressed in stromal cells during the early stages of intramembranous bone development and in osteoblasts, and it is stored in the extracellular matrix in bone tissue [151]. It activates extracellular signal-regulated kinase 1/2 (ERK1/2) signaling and promotes the acetylation and stabilization of transcription factor Runx2 associated with osteoblastogenesis [152]. This GF is an effective therapeutic agent with a high affinity for glycosaminoglycan heparin-binding sites on cells. On the other hand, it is a fairly unstable protein with a short half-life [95,96]. The stabilization of bFGF and more efficient binding to receptors can be improved by the addition of inorganic polyphosphate. An in vivo and in vitro study has shown the upregulation of osteocalcin, osteopontin, and collagen type 1 messenger ribonucleic acid (mRNA) expression in MC3T3-E1 osteoblast-like cells and MSC, respectively, in the presence of bFGF and polyphosphate-modified interconnected porous hydroxyapatite complex. The presence of polyphosphate significantly affects tissue regeneration and cell growth compared to GFs alone [89,92].

**Figure 5:**
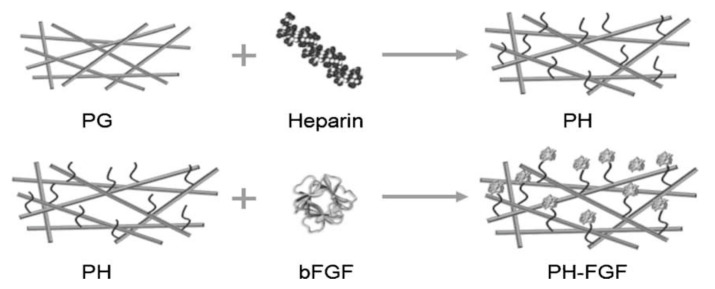
Schematic representation of the heparinization and immobilization of bFGF. PG represents the PCL/gelatin fibrous matrices cross-linked with genipin, PH represents PG fibers conjugated with heparin, and PH FGF represents a coating with immobilized bFGF. Reprinted with permission from [155], John Wiley and Sons, 2011.

Although titanium-based implants with poly(ethylene glycol) (PEG) coatings have a positive effect on cell adhesion, an in vivo study has shown that the incorporation of bFGF promotes bone tissue maturation, which is characterized by the presence of numerous chondrocytes and hypertrophic chondrocytes. Nevertheless, bFGF has not been shown to induce bone healing in closed fractures in animal models [98,99]. PEG has been shown to be a promising material for the incorporation of GFs, as it covers the immunogenicity of biomolecules and provides protein stability and protection against proteolytic degradation [153]. In addition to the stability of the GF in the coating, the ability of the coating material to bind and uniformly deliver a certain suitable concentration is important. The required concentration of bFGF varies depending on the cell type, as shown in an in vitro study with baby hamster kidney cells (BHK-21) and the osteosarcoma MG-63 cell line. In MG-63 and BHK-21 cells, proliferation increased with increasing bFGF concentration between 1–10 ng/mL and 1–100 ng/mL, respectively. However, proliferation was decreased at higher concentrations [91]. Since bFGF can induce BMP-2 expression [90,94], the upregulation of BMP-2 was observed in MG-63 cells, suggesting the possibility of osteoblast activation with an adequate concentration of bFGF [91]. Moreover, another in vivo study demonstrated the osseointegrative effect of implants with bFGF in the coating, and confirmed efficacy with an in vitro study as well. Additionally, the incorporation of bFGF in coatings by means of a solution of Dulbecco’s phosphate-buffered saline and calcium chloride (CaCl_2_) affected the surface of the implant, which implies greater homogeneity, and bFGF also retained its bioactivity [97]. Stability and protection from the inactivation of bFGF can be achieved by binding bFGF to heparin [154,155]. It has been shown that the zero-order kinetics of bFGF release result from the chitosan coating, which includes heparin-containing polyelectrolyte complex NPs, with the NPs composed of heparin and chitosan. Solutions of heparin and chitosan were individually prepared with 0.1 M acetate buffer and then mixed in a volume ratio of 4:1 to form a complex that aggregated into NPs due to mixing. The polyelectrolyte complex NPs controlled the release of bFGF for up to 30 days. With the addition of a polyelectrolyte multilayer on top, composed of N,N,N-trimethyl chitosan and heparin, in which no bFGF release was detected, the polyelectrolyte complex of heparin and chitosan NPs is a promising additional component of the chitosan coating [87]. The content of immobilized heparin, which effectively binds bFGF, can be controlled by mixing PCL and gelatin with different electrospinning volumes (6, 12, and 24 mL) to obtain fibrous matrices of various thicknesses. This results in a non-woven matrix that allows controlled bFGF release (Figure 5). Compared to the coating without heparin, which demonstrated an initial burst release of approximately 94% bFGF in 7 days, bFGF was released slowly for a period of 56 days [155].

In designing a bioactive coating with incorporated therapeutic agents, such as GFs, polymers usually serve as their carriers. However, polymer-based bioactive coatings can reduce the favorable surface roughness of anodized titanium implants and can inhibit contact between the bone and implant surface. In an in vitro study in which anodized titanium discs were coated with bFGF-loaded PLGA NPs, it was reported that the coating significantly contributed to cell differentiation and proliferation. This positive effect was also confirmed by an in vivo study in which the average osseointegration values were 70.1% with the coating and 47.1% without the coating [88]. The problem with GFs is their instability and short half-life. The bioactivity of bFGF, which is important for the pluripotency and self-preservation of stem cells, falls below 50% within 12 h. Protection against the denaturation of bFGF and the preservation of its bioactivity in the physiological environment can be achieved with heparin. The addition of heparin to bFGF affects the formation of complexes between bFGF and the fibroblast growth factor cellular membrane receptors [86,156,157]. However, the rapid release of bFGF from such a coating has been achieved using a layer-by-layer coating technique [86]. NPs such as superparamagnetic iron oxide can also be used for the controlled release of GFs [84,158]. NPs coated with human serum albumin have been found to effectively conjugate bFGF, resulting in a more controlled release of bFGF [84]. Effective binding and sustained release of bFGF can be achieved by combining D-RADA16 polypeptide hydrogels with nano-hydroxyapatite/polyamide 66. D-RADA16 is a biocompatible peptide composed of arginine (R), alanine (A), and aspartate (D), self-assembled into network structures. Together with nano-hydroxyapatite/polyamide 66, D-RADA16 inhibits the burst release of bFGF, thereby reducing cellular deformation. However, due to the complete degradation of the polypeptide after 7 days, there was no significant difference in the amount of bFGF released compared to the release of bFGF incorporated in nano-hydroxyapatite/polyamide 66 alone [85]. The composition of the multifunctional coating of hydroxyapatite and bFGF can be improved by the addition of the antibiotic kanamycin. This composite coating has been shown to inhibit biofilm formation in vitro without harming murine cells. A corrugated outer layer and rod-shaped morphology were also observed, which contribute to better cell adhesion [83].

### 2.4. The Wingless-Type MMTV Integration Site Family Member 3A (Wnt3A)

Wnt proteins are a group of signaling molecules that act as GFs. By stabilizing the β-catenin protein, they mediate processes in bone cells, such as osteoblast attachment, differentiation, and apoptosis [159]. The Wnt3A GF improves the cell density of osteoblasts on scaffolds, activates the β-catenin pathway, and increases the expression of β-catenin after treatment under diabetic conditions, leading to improved cell adhesion, cytoskeletal organization, and cell morphology [100]. Enhanced bone formation was also observed in in vivo studies in mice in the presence of Wnt3A. Wnt3A incorporated into a collagen sponge caused a significant difference in healing of the calvary bone after 7 days of treatment compared to the control group in the absence of Wnt3A [101]. Another possibility for achieving the controlled release of Wnt3A is to coat supermagnetic iron oxide NPs with a temperature-sensitive polymer such as poly(N-isopropylacrylamide). The incorporation of a GF such as Wnt3A into the NPs, which are cross-linked in poly(N-isopropylacrylamide), allows for the controlled release of the GF. In this case, the controlled release of Wnt3A occurs when the magnetically heated NPs trigger release from the temperature-sensitive polymer. This increases MSC proliferation and prevents an overdose, which can create a toxic environment for cells and cause more harm than good. The advantage of such application is the release of Wnt3A at the desired time, and its inactivity until the release occurs. The disadvantage, however, is the possible harmful effect of hyperthermia, which can occur as well [158]. Although Wnt3A and its incorporation into bioactive coatings have not yet been studied in detail, Wnt3A has been shown to be an effective GF that can simultaneously inhibit osteoclast activity. Collagen sponges serve as carriers of Wnt3A GF for local application. Wnt3A upregulates distal-less homeobox-5, which modulates Runx2, thereby affecting osteoblast differentiation. During the inflammatory state in bone tissue, there may be increased osteoclast activity, which can be inhibited with Wnt3A by activating the Wnt signaling pathway [102].

### 2.5. Insulin-Like Growth Factor-1 (IGF-1)

**Figure 6:**
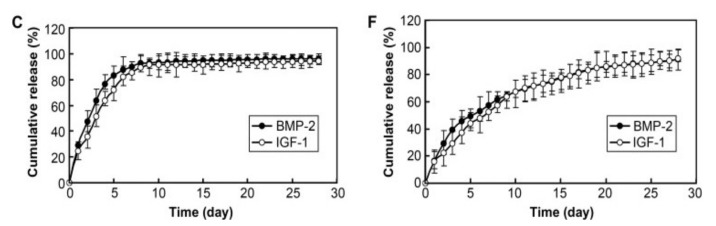
Release profiles of BMP-2 and IGF-1 (**C**) from a scaffold containing adsorbed GFs and (**F**) from a scaffold containing microparticles with loaded GFs. Reprinted (adapted) with permission from [106], Elsevier, 2009.

The role of bioactive coatings as carriers of therapeutic agents is to control their release. Burst release can be prevented by encapsulating GFs in glycidyl methacrylate dextran/gelatin microparticles and incorporating them into the scaffold instead of loading them by adsorption. It has been shown that 90% of IGF-1 and BMP-2 loaded by adsorption can be released in less than 7 days. In contrast, burst release was prevented, and the period of GF release from a scaffold containing microparticles was extended to 21 days (Figure 6). Moreover, the incorporation of microparticles into coatings enabled the delivery of multiple GFs [106].

Another possible carrier for the dual delivery of IGF-1 and BMP-2 is a complex coating of PLGA/hydroxyapatite with polydopamine. The addition of polydopamine contributes to hydrophilicity, which is beneficial for the binding of proteins such as GFs [103]. Specifically, it has been found that moderately hydrophilic substrates provide better cell proliferation, adhesion, and growth than hydrophobic or highly hydrophilic ones [160,161]. An in vitro study on MC3T3-E3 cells showed that cell adhesion was better with coatings of adsorbed IGF-1 or IGF-1 and BMP-2 together than BMP-2 alone. On the other hand, an in vivo study on rabbits showed that scaffolds with immobilized BMP-2 or BMP-2 and IGF-1 together had a significantly higher healing rate and higher osteoconductivity compared to scaffolds with immobilized IGF-1 alone. These results suggest that not only is polydopamine-assisted surface modification an effective method for immobilizing GFs [103], but that incorporating multiple GFs provides a better capacity to promote bone regeneration than using them alone [103,106].

IGF-1 is very commonly used in combination with another GF, such as transforming growth factor-β1 (TGF-β1). Locally delivered IGF-1 and TGF-β1 incorporated into a biodegradable PDLLA coating showed a synergistic effect on fracture healing. This combination appears to enhance the mechanical fixation and osseointegration of Ti6Al4V implants and is comparable to hydroxyapatite [107,109,110]. The biodegradable PDLLA coating serves as a carrier for several GFs simultaneously while also affecting the mechanical stability of the metal implant and retarding the process of intramedullary insertion. An in vivo study on rats confirmed the importance of incorporating GFs into such coatings. Fractured tibiae healed in 50% of cases after 28 days and in 90% of cases after 42 days with the presence of IGF-1 and TGF-β1 in the coating. However, in the absence of GFs, the fracture was still evident in the bone after 42 days. It should also be noted that a PDLLA coating was found to enable the continuous release of 80% of incorporated GFs within 42 days [162]. The synergistic effect of these two GFs was demonstrated in an in vivo study on minipigs. The PDLLA coating itself promoted the healing process of a bone fracture, and the incorporation of IGF-1 and TGF-β1 further accelerated the healing process [108]. Since GFs influence cell proliferation and differentiation, the balance and manipulation of these is very important for achieving the desired rate of proliferation and degree of differentiation. An in vitro study with the osteoblast cell line hFOB 1.19 revealed the release of approximately 50% of incorporated IGF-1 and TGF-β1 from the PDLLA coating in the first 48 h. Furthermore, a 10 µm thick PDLLA coating was found to enable the release of 22% of IGF-1 and 18% of TGF-β1 within 1 hour, which in turn stimulated differentiation and reduced the proliferation of osteoblasts [163].

Polyetheretherketone (PEEK) is also a promising biomedical material for orthopaedic and dental applications due to its good mechanical properties and very low cytotoxicity. The immobilization of the GFs IGF-1 and BMP-2 on porous PEEK with polydopamine as a bioactive coating resulted in the adhesion and proliferation, extracellular matrix (ECM) secretion, and osteo-differentiation of MC3T3-E1 cells being increased due to the coating with polydopamine. This means that the combination of IGF-1 and BMP-2 can promote the attachment and proliferation, ECM secretion, and osteo-differentiation of MC3T3-E1 cells and significantly increase the bioactivity of PEEK. The disadvantage of PEEK is its low bioinertness and poorer osteo-conductivity in terms of compatibility with bone tissue, which can be improved by the application of bioactive coatings [105]. The use of PEEK can be improved through sulfonation and modification with graphene oxide, as this increases bioinertness, improves cell adhesion and proliferation, stimulates bone mineralization, and results in increased activity of the ALP enzyme, signifying the presence of osteoblasts [104]. IGF-1 plays a crucial role in maintaining bone density, which deteriorates with age due to decreasing levels of IGF-1 in the blood [111]. IGF-1 can be loaded into polyelectrolyte multilayers, as shown in an in vitro study on bone marrow mesenchymal stem cells. A titanium implant was coated with IGF-1-loaded gelatin/chitosan and polyethyleneimine as an excitation layer. Higher concentrations of IGF-1 in such a system have been shown to have a beneficial effect not only on the hydrophilicity of the coating but also on cell proliferation and osseointegration [164].

### 2.6. Vascular Endothelial Growth Factor (VEGF)

The success and rate of healing depends on the blood supply to the tissue, as cells are supplied with nutrients and oxygen through the bloodstream. The development of blood vessels is important for good blood flow in the initial phase of healing, the formation of which is induced by VEGF [128,165]. VEGF not only promotes angiogenesis but also stimulates mRNA expression of ALP and Runx2, which are critical for bone mineralization and osteoblast proliferation [114]. A coating of carboxymethyl chitosan or hyaluronic acid with incorporated VEGF has an antibacterial effect by inhibiting bacterial adhesion, while the presence of VEGF promotes osteoblast functions [119]. The presence of multiple GFs is desirable for the successful regeneration and integration of the implant. The co-incorporation of BMP-2 and VEGF into a coating of chitosan and sodium hyaluronate on the hydroxyapatite scaffold caused the increased expression of osteopontin and collagen type 1, which are required for healing. Approximately 40% of the BMP-2 and VEGF were released from the coating in the first day and 80% after 14 days [116]. An in vivo study on rats also demonstrated the synergistic effect of the simultaneous inclusion of multiple GFs. In the area of bone damage, more new bone tissue was formed in the presence of a combination of BMP-4, VEGF, and bFGF than in the presence of a single GF. Collagen fibers, among others, were detected only in the presence of the combination of all three GFs [98]. The potential use of a mixture of PLA and β-tricalcium phosphate as an implant material was investigated in an in vivo study on mice using a porous scaffold composed of a combination of PLA and β-tricalcium phosphate. With BMP-2 and VEGF incorporated into the collagen coating, an implant inserted into the muscle caused increased blood vessel formation [117]. The combination of BMP-2 and VEGF has an effect on the increasing bone volume density, as shown by an in vivo study on domestic pigs. On the other hand, the bone-implant contact was not ideal in an implant in which both GFs were incorporated. It was concluded that the simultaneous presence of BMP-2 and VEGF on the surface affects the recruitment of osteoprogenitor cells and integrin-mediated osteoblast attachment [125].

**Figure 7:**
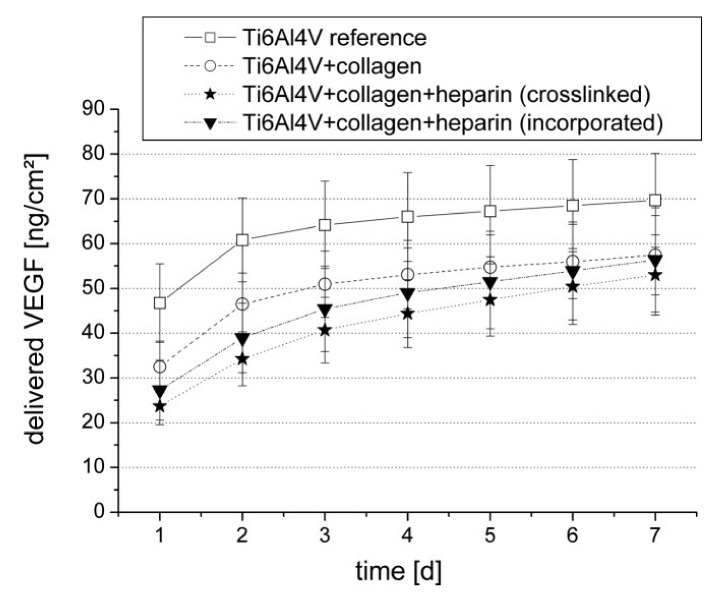
The release profile of VEGF from Ti6Al4V implant material, implant material with a collagen coating, implant material with a heparin-cross-linked collagen coating, and implant material with a heparin-incorporated collagen coating. Reprinted with permission from [120], John Wiley and Sons, 2006.

Coating with chitosan alone, chemically bonded to a titanium surface by silane glutaraldehyde linker molecules, does not provide the prolonged release of VEGF. Over a period of 3 days, 90–95% of the VEGF was released from this kind of coating [115]. This period is sufficient for the activation of angiogenesis by VEGF [130], although some studies suggest that the prolonged release of lower concentrations is better for osseointegration [129,166]. The amount of VEGF released over time depends on the efficiency and type of immobilization in the coating (Figure 7). The binding of VEGF to heparin in a collagen coating provides prolonged release, and the presence of heparin increases mitogenic activity and cell proliferation compared to a collagen coating alone [120].

An in vivo study in dogs showed the importance of blood flow for bone regeneration, with VEGF playing a crucial role by significantly accelerating vascularization and mineralization around the implant. The porosity of the implant into which the GF is incorporated is also important, as it allows osteoblasts to proliferate within the pores, increasing both osseointegration and vascularization [126]. VEGF incorporated into a PCL coating for Ti6Al4V alloy and magnesium scaffolds has been shown to be biocompatible, stimulating cell growth and proliferation as well as accelerated angiogenesis [118,121]. In contrast, in the absence of VEGF, a PCL coating was found to impair angiogenesis and achieved worse results than a pure porous titanium implant. Despite cell proliferation and angiogenesis, the major disadvantage was undesired burst release, as most of the VEGF was released from the coating within the first three days [118]. However, the desired controlled release could be achieved by incorporating VEGF nanoreservoirs into the PCL coating. Nanoreservoirs of VEGF incorporated into chitosan NPs not only provided sustained release, but also enhanced cell reorganization and thus vascularization [124]. Prolonged release of VEGF for up to 28 days, excluding the initial burst release, was enabled by a coating made of silk fibroin. This made the surface of the implant materials, such as ultra-high-molecular-weight polyethylene, more hydrophilic [127]. In addition, silk fibroin, with its surface roughness, improved the adhesion and proliferation of cells that had difficulty settling on the implant due to chemical inertness and the poor interfacial adhesion of the implant itself [122,127]. The immobilization of VEGF on hydroxyapatite coatings can be improved by ion substitution with silicone, which at lower levels alters the surface of the implant in a manner suitable for osteoblasts. Such a coating was found to accelerate angiogenesis and healing, but excessive amounts of silicone led to impaired cell adhesion to the implant surface [113]. VEGF is indispensable in silicone and hydroxyapatite coatings because the coating itself does not adequately promote the proliferation of pre-osteoblasts. In contrast to pre-osteoblasts, the presence of silicone significantly decreases the proliferation of endothelial cells [123].

### 2.7. Platelet-derived growth factor BB (PDGF-BB)

As mentioned above, IGF-1 is a growth factor that enhances proliferative, osteoinductive, osteoconductive, and integration effects in combination with other growth factors. Also, in combination with recombinant human PDGF-BB (rhPDGF-BB), its effect in the healing process of a cortical wound in the tibiae of Yucatan miniature pigs was found to be greater than with a single growth factor, with rhPDGF-BB and IGF-1 being delivered via syringes to the wound site [134]. The same positive effects of PDGF-BB and IGF-1 were found in the first human clinical trial, involving 38 individuals possessing bilateral osseous periodontal lesions [112]. Moreover, a combination of three growth factors, TGF-β, IGF-1, and PDGF, achieved significantly higher bone matrix apposition than a single GF or a combination of two [139]. The same positive, enhancing effects were found in combination with synthetic peptides such as AC-100 (Dentonin™), thrombin-related peptide TP508 (Chrysalin™), and p15 (Pepgen™) [167]. On the other hand, some studies have shown increased collagenase enzyme activity [132] and a resorption effect from PDGF-BB on bone tissue [141].

Since an optimized artificial joint requires multiple components, such as the implant itself, the bioactive coating, and the therapeutic agent, the success of PDGF-BB integration is important. In vivo studies in beagle dogs have shown that dental metal implants coated with hydroxyapatite are suitable carriers of rhPDGF-BB. More newly formed bone tissue was observed between the implant and bone in implants with absorbed rhPDGF-BB than in the absence of rhPDGF-BB [137]. Similar positive effects were observed in rats with titanium implants used. During the first weeks, the depth of connective tissue penetration into the implant grooves in the case of rhPDGF-BB was significantly increased compared to the implant without a growth factor and with enamel matrix derivative (EMD) incorporated. However, after four weeks, the depth of tissue penetration into the implant threads continued to increase in EMD-coated implants, while it stagnated in the case of rhPDGF-BB [168]. PDGF-BB with β-tricalcium phosphate has a positive effect on intraosseous periodontal healing defects [138]. In addition to β-tricalcium phosphate, the equine-derived bone matrix is also suitable as a carrier for rhPDGF-BB, as shown in a study of 32 adult periodontitis patients. Dental implants with an equine-derived bone matrix carrier performed even better than β-tricalcium phosphate, as the implant was better integrated. Nevertheless, both materials were able to maintain the crestal bone height [140]. On the other hand, the osteoinductive effect of rhPDGF-BB was also investigated in mice, where gelatin capsules were filled with uncoated particulate hydroxyapatite β-tricalcium phosphate (HA-TCP), EMD-coated HA-TCP, or rhPDGF-BB-coated HA-TCP. Although no osteoinductive effect was observed despite the inclusion of rhPDGF-BB or EMD, the soft tissue response showed biocompatibility, as no side effects occurred [169].

PLLA is deemed to be an appropriate carrier to achieve controlled release of GFs. It has been shown that, by coating polyglycolic acid meshes with PDGF-BB-dissolved PLLA, controlled release of PDGF-BB can be achieved with the addition of bovine serum albumin, releasing 2 ng PDGF-BB per day from an initial mesh volume of 100 ng [170]. The hydrophobicity of PLLA can be modified with chitosan. It has been reported that the addition of chitosan to a coating increased hydrophilicity, wettability, and biocompatibility and enabled controlled release of PDGF-BB, which in turn resulted in accelerated bone healing [136]. Prolonged release over 8 weeks was achieved in a study in which the PCL scaffold was improved by the addition of type 1 collagen and hydroxyapatite into which PDGF-BB had been incorporated. Such a bone-mimetic electrospun scaffold has a greater capacity to adsorb and release PDGF-BB than a scaffold composed of PCL alone [171]. Anodized titanium scaffolds functionalized with dopamine and heparin were found to allow the prolonged release of a combination of BMP-2 and PDGF-BB over 28 days without a cytotoxic effect on MG63 osteoblast cells [172].

As well as implant coating, suture coating with rhPDGF-BB has shown accelerated healing. In an in vivo study in sheep, sutures were coated with rhPDGF-BB with the gelatin dip-coating technique, with rhPDGF-BB improving histological results and tendon healing compared to suture-only controls [135]. In another in vivo study in sheep, type 1 collagen matrixes with 75 µg and 150 µg of incorporated rhPDGF-BB showed accelerated healing and better tendon–bone connections [133]. A similar study was performed with rats in which rhPDGF-BB was incorporated into a collagen scaffold. The presence of rhPDGF-BB did not result in increased repair, which could have been due to insufficient concentration of rhPDGF-BB. However, increased cell proliferation was detected [131].

### 2.8. The Influence of Coating Materials and the Indirect Involvement of GFs

To a lesser extent and not as effectively as in the presence of growth factors, cells can be manipulated to proliferate and differentiate with different materials. In addition to GFs included in bioactive coatings, it is also important to control the function of the GFs already present in the cells. Some implant and coating materials are capable of inducing the expression of genes encoding a sequence for GF synthesis. In this manner, the cells ensure the supply of GFs by themselves, which results in better adhesion and osseointegration. Materials that have the ability to manipulate cells include metals [173], ceramics [174], synthetic polymers [175], and natural materials [176]. The following sections cover coating materials.

#### 2.8.1. Synthetic Coatings

One example of a coating material with an effect on proliferation and differentiation is the complex polymer PEG-block-PCL with the addition of lactoferrin. The inclusion of lactoferrin in the biodegradable synthetic polymer PEG-block-PCL was found to promote cell adhesion, positively modulate morphology, and enhance cell proliferation, which are associated with an increased capacity for osteogenic differentiation and the adaptation of cells to surface properties [177].

Hydrogels have great potential for mimicry of the cellular microenvironment and are therefore suitable as coatings of artificial joints. Platelet-rich plasma (PRP) is an autologous concentrate that has been shown to improve the healing process due to its high level of natural GFs. When combined with hydrogel, i.e., methacryloyl gelatin, PRP induces positive interactions between the implant and fibroblasts, highlighting the potential use of PRP with hydrogels for both artificial joints and meshes for hernial repair [178].

Promising materials also include bioactive glass, which has the ability to enhance the bioactivity of gelatin [179] and PLGA [180]. Bioactive glass with the addition of silver ions has been shown to have a cytotoxic effect in the presence of peripheral blood mononuclear cells (PBMCs) [181], while bioactive glass scaffolds coated with PCL in the presence of graphene nano-powder have been shown to be biocompatible with mouse BMSCs [182]. The latter can be used to develop a bioactive coating. The authors state that their in vitro studies need to be repeated with the incorporation of GFs and other therapeutic agents into the coatings in order to improve implant performance. Such system should then be studied in vivo. Among other materials, bioactive glass beads have been found to have a positive effect on VEGF expression in fibroblasts, as the expression was four times higher than in the absence of bioactive glass. It has also been shown that there is no significant difference in the cellularization and vascularization of bone, as the tissue itself has growth-promoting properties [183].

Mesoporous bioactive glass obtained with the sol-gel method showed high bioactivity in vitro and could bind drugs and GFs very well due to its flexible pore size [184]. In vitro bioactivity and possible drug delivery were discussed in a study using titanium dioxide (TiO_2_)-based scaffolds coated with mesoporous calcium silicate beads obtained with the sol-gel method. It was found that 80% of ibuprofen was released from the coating in the presence of a TiO_2_ scaffold within 24 h. On the other hand, in the absence of a TiO_2_ scaffold, the same amount of ibuprofen was released within 48 h. All of the ibuprofen was released within 14 days, indicating the potential for the use of this type of coating with the incorporation of GFs [185]. Faster bone regeneration in the area of the injury or in the area of contact with an orthopaedic implant can be achieved by modifying skeletal bioactive borosilicate glass with mesoporous bioactive glass. Mesopores with a size of 4 nm and macropores provide an excellent basis for cell seeding and stimulate cell proliferation and differentiation, as shown by an in vitro study with human BMS cells and an in vivo study that did not use GFs [186]. It is very likely that better proliferation and differentiation can be obtained by immobilizing GFs, but additional research would be required to confirm this.

The importance of the presence of GFs in coatings was demonstrated by an in vivo study with sheep that used bioactive ceramics as a scaffold and incorporated zirconium into the crystalline structure of calcium silicate, known as baghdadite. The efficiency of bone integration with the implant was the same for uncoated implants and implants with a PCL coating containing bioactive glass NPs [187]. Such materials have a beneficial effect on proliferation, angiogenesis, and implant acceptance, but GFs are key to improving the response of bone tissue to the implant [88].

#### 2.8.2. Coatings Based on Naturally Occurring Compounds

**Figure 8:**
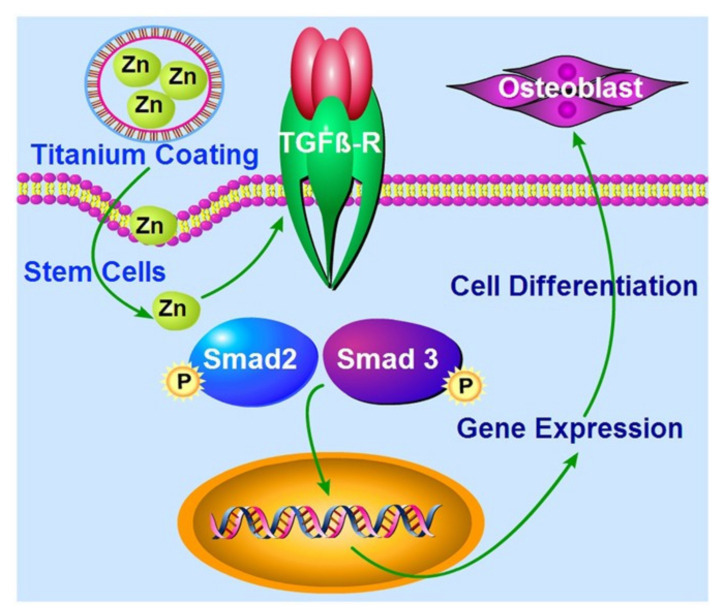
Schematic representation of the probable molecular mechanism of Zn-modified coatings, inducing the expression of genes required for cell differentiation from BMP-PC to osteoblasts via the TGF-β/Smad pathway. Reprinted with permission from [191], Springer Nature, 2017.

Biodegradable coatings containing calcium phosphate and hydroxyapatite have been shown to stimulate cell proliferation and the expression of BMP-2, VEGF, and transforming growth factor-β2 (TGF-β2) genes and to enhance the regulatory effects of BMP-2, VEGF, and TGF-β2 at various stages of reparative osteogenesis in vivo [188]. In the absence of exogenous osteogenic factors, the osteogenic differentiation of bone stem cells can be induced and maintained by coating the Ti6Al7Nb implant with hydroxyapatite and, to a lesser extent, with silicate titanate. Such a cell response is due to the chemical and topographic properties of the coatings and the implant [189]. In a comparative in vitro study, biocompatibility was tested with three types of titanium-based alloy implants: Ti6Al7Nb implants with a total porosity of 25%, used as a control; implants infiltrated with hydroxyapatite (Ti-HA) through the sol-gel method; and silicatitanate implants. Osteoblast response was found to depend on the type of implant and growth conditions. Silicatitanate implants maintained osteoblast adhesion and promoted differentiation through the increased production of collagen and non-collagenous proteins. In contrast, the Ti-HA had a lower ability to induce cell adhesion and proliferation but a greater ability to promote early mineralization, although the addition of BMP-2 and TGF-β1 in the differentiation medium did not improve the mineralization process [190]. Calcium phosphate and hydroxyapatite coatings on a Ti6Al4V implant have been shown to improve the bone integration of metal implants compared to uncoated implants, and osteogenic differentiation can be achieved with the addition of zinc salt. It was found that the Zn(NO_3_)_2_·6H_2_O:Ca(NO_3_)_2_·4H_2_O:(C_2_H_5_O)_4_Si complex, at a ratio of 0.3:1:1 in the coating, was sufficient to induce an increased expression of genes associated with the TGF-β/Smad pathway, which is critical for osteogenic differentiation (Figure 8). When a ratio of (<0.3):1:1 (i.e., a lower amount of Zn(NO_3_)^2^·6H_2_O) was tested, it did not provide sufficient gene expression to trigger osteogenic differentiation [191].

Moreover, a combination of calcium phosphate, chitosan, and hyaluronic acid accelerated early in vitro stem cell differentiation into an osteoblast-like lineage and also accelerated the maintenance and induction of paracrine secretion of VEGF [192]. In this regard, it is highly likely that the effects of the coating could be further enhanced by the inclusion of GFs. In an in vivo study in mice, the difference in response to uncoated Ti6Al4V implants with a bioactive coating of calcium phosphate and hydroxyapatite was investigated. The authors demonstrated the importance of bioactive coatings, as a positive effect from the coating was found with regard to the expression of VEGF and TGF-β2, while, in terms of angiogenesis, the coating resulted in the acceptance of the implant [193]. In addition to calcium ions, magnesium ions are also used to enrich the surfaces of titanium (grade 4) implants and TiO_2_ coatings. Magnesium ions are important for protein formation, the expression of GFs, and the deposition of bone minerals on implant surfaces. However, coating decomposition can result in rapid ion release and consequently lower adhesion efficiency [194].

A collagen membrane can also be used as a guiding membrane for bone regeneration. Functionalization with a hybrid coating consisting of calcium phosphate, chitosan, and hyaluronic acid by the simultaneous spraying of interacting species improved the mechanical properties of the implant compared to the non-functionalized implant [195]. The effectiveness of such a system might even be improved by the inclusion of a specific GF affecting cell adhesion and differentiation around the implant. Very low cytotoxicity was observed in an in vitro study in which the microRNA (miRNA) miR-29b was encapsulated in nanocapsules in an O-carboxymethyl chitosan coating. This was not only favorable for cell adhesion and growth, but also provided sufficient efficiency for miRNA transfection and osteoinduction, resulting in a significant improvement in bone regeneration on the titanium alloy, which had a bioinert surface [196]. In an in vivo experiment on rabbits, a histological examination showed that a scaffold of strontium-enriched polyphosphate coated with polydopamine and silk fibroin could effectively accelerate bone mineralization and regeneration. In addition, an immunohistological study showed increased secretion of VEGF and bFGF from the host cells [197].

The addition of simvastatin to alginate improved the effectiveness of a TiO_2_ coating by increasing the expression of osteoblast-related genes in MSCs in the presence of the aforementioned substance. In the case of human adipose tissue-derived mesenchymal stem cells (hAD-MSCs), a statistically significant increase in mRNA expression of collagen type 1 α1, ALP, osteopontin, osteocalcin, and vascular endothelial growth factor A (VEGFA) was observed due to simvastatin exposure without inducing a cytotoxic effect. Moreover, the secretion of osteopontin, osteoprotegerin, VEGFA, and osteocalcin proteins was significantly increased in hAD-MSCs grown on a pad containing 10 nM simvastatin, thus confirming osteogenic differentiation [198]. The effect of GFs of the TGF-β family may outperform that of decellularized cartilage derived from porcine articular cartilage. An in vitro experiment on rat bone marrow-derived mesenchymal stem cells demonstrated the proliferation and increased expression of the genes responsible for bone tissue regeneration [199]. A gel derived from autologous blood, consisting of PRP, was found to be a good source of GFs such as TGF-β, VEGF, and IGF-1 [200]. A plasma-coated titanium implant has also shown promise in promoting proliferation [201]. However, PRP also contains thrombospondin-1 and other proteins that may inhibit proliferation, cell adhesion, and angiogenesis at higher PRP concentrations [200].

## 3. Conclusions and Further Perspectives

By applying bioactive coatings incorporating therapeutic agents, i.e., bone-inducing growth factors (GFs), implants can be improved such that they are more readily accepted by bone tissue, which means the life of the implant is prolonged, and this consequently prevents revision surgeries. The GFs reviewed in this work are the most commonly used and investigated in the context of bioactive coatings. Bone first interacts with the implant surface; therefore, appropriate surface modifications can improve or introduce new implant properties in terms of bioactivity, osteoconduction, and osteoinduction. Due to the need to extend implant longevity and promote bone healing, GFs are proving to be a key component of bioactive coatings. The surface of the implant should mimic the bone surface or tissue, which can be achieved by incorporating GFs such as bone morphogenic proteins (BMPs), basic fibroblast growth factor (bFGF), vascular endothelial growth factor (VEGF), wingless-type MMTV integration site family member 3A (Wnt3A) protein, insulin-like growth factor-1 (IGF-1), and transforming growth factor-β (TGF-β). Among all the GFs, BMPs are the most widely investigated because they strongly induce neovascularization and positively influence angiogenesis during tissue-engineering regeneration of large bone defects.

The main advantage of the local delivery of GFs from bioactive coatings is the avoidance of systemic drug delivery and the direct delivery of the drug to the target cells. Local delivery of GFs increases their effectiveness compared with systemic treatment. However, there are also some disadvantages associated with local delivery, e.g., possible burst release. In the event that the release kinetics follow a burst release pattern, it is necessary to reduce the concentration of the incorporated GFs in the bioactive coating. Moreover, burst release results in an undesired short release time. Furthermore, high local GF concentrations are also u desirable due to the possible cytotoxic effect. On the other hand, low concentrations of GFs are usually not sufficiently effective to induce the desired cell proliferation and differentiation in the long run. Therefore, the development of a coating with the desired release kinetics for the GFs is currently being sought.

The determination of the required concentrations of GFs in bioactive coatings involves the determination of the concentration necessary to achieve a significant difference in the presence and absence of growth factors and of the varying concentrations in either the expression of genes—as characteristic of cell proliferation and differentiation into osteoblasts in vivo and in vitro—or in the forces necessary to separate the integrated implant from bone tissue in vivo. Determination of growth factor concentrations in the coating or on the scaffold surface in vitro and in vivo is usually done by calculating the amount of incorporated growth factor per scaffold surface or the amount of incorporated growth factor per volume of solution in which the scaffold is incubated. However, in vivo in larger animals, growth factor concentration is most commonly calculated as the amount of growth factor in the volume of solution in which the implant is incubated. Generally, the amount of growth factor incorporated is calculated as the difference between the total growth factor concentration in the solution and the concentration of the growth factor remaining in the solution after incubation of the implant or scaffold.

In in vivo research, comparisons of bare implants and implants with growth factors in terms of their osteoconduction, osteoinduction, and osseointegration are often performed. The success of integration in terms of the proportion of newly formed vessels, the proportion of bone tissue, and the quality of newly formed bone tissue (bone cells and extracellular matrix) can be assessed more quickly in smaller animals due to their small size and faster healing. Despite these advantages, the impossibility of measuring their mechanical properties prevents the translation of these implants into clinical practice. Larger animals have an advantage in this regard as they are closer to human proportions in terms of mass and the forces required for movement. In vivo studies on larger animals provide information on the osseointegration and mechanical strength of the implant, which means that it is possible to determine the loads that the implant integrated into the bone can withstand in order to ensure that the two do not separate. On the other hand, such studies require much more time, with larger animals also representing a more significant financial burden for the research.

To determine the amount of growth factors required in tissue engineering, it is also important to compare endogenous (i.e., synthesized continuously in the organism) and exogenous (i.e., coating-derived) growth factors. Unfortunately, the literature still does not adequately emphasize the difference in the effects of these growth factors, as the effects of growth factors on osteoconduction, osteoinduction, and osseointegration are emphasized in comparison to the bare implant or scaffold, with the amount of endogenous growth factors often not determined. Due to these endogenous growth factors, the main problem is to determine the effects on long-term implantation. Further research is needed to make such an assessment. The correct anatomical location of bone formation is crucial for the healing process of damaged bone. Therefore, the mechanical and biochemical environment of an orthotopic bone model should not be neglected, with growth factors activating the various signaling pathways involved in this process.

The ideal release kinetics of GFs are slow and continuous. For successful osseointegration, it is important that the GFs are released simultaneously with the degradation of the bioactive coating. In such a manner, the implant can be successfully integrated into the bone tissue. The shortest release time reported, due to burst release, was in the range of a few hours. On the other hand, a maximum release time of 56 days was also reported. To achieve prolonged release, it is often necessary to increase the dose of growth factors contained in the coating. Since growth factors are relatively expensive therapeutic agents, maximizing the dose results in higher costs, making further clinical translation difficult.

The adequacy of in vitro release kinetics in in vivo models can be assessed in two steps. First, sequential measurements must be made of the amounts of growth factor released from the in vitro coating into the PBS during incubation under physiological conditions. These are then transferred to in vivo models by macro/microscopic examination of the coated implanted tissue. Micro-computed tomography is one of the most commonly used methods for determining integration success in an in vivo model, as it can determine the quality and quantity of newly formed bone along with the bone mineral density and the ratio of bone volume to total volume. In order to achieve long-lasting and controlled release, it is necessary to include other therapeutic agents, such as heparin and fibronectin, in bioactive coating development, as they specifically bind GFs, thus allowing controlled release, prolonging the bioactivity of GFs, and increasing their effectiveness. Moreover, the incorporation of multiple GFs into a bioactive coating induces a further synergistic effect. It has been reported that the incorporation of several GFs into a bioactive coating is more effective in treatment than the incorporation of a single one. Since different GFs act on different signaling pathways and thus increase the proliferative and differentiating effects, i.e., the quality of the newly formed bone tissue around the implant, the probability of the need for revision surgeries is reduced. In the future, all bioactive coating modifications will allow the design of implants and bioactive coatings according to the needs of individuals. The selection of the appropriate GF or a combination of several GFs together with coating and implant materials will significantly affect the development of personalized medicine, which will subsequently reduce the number of revision surgeries. It will also allow the customized design of implants according to the specificities of the most demanding group of patients, namely those with osteoporosis.

## Figures and Tables

**Table 1:** Advantages and disadvantages of the GFs considered in this review (*—not yet reported for bioactive coatings for implants).

GF	Study	Advantages	Disadvantages
BMP-2	In vitro	Enhances proliferation and osteogenesis [48,49,50,51,52,53,54,55,56,57,58,59]	Short half-life [19]; toxic at 200 ng/ml [60]
	In vivo	Faster healing and more newly formed bone tissue [48,49,50,52,54,56,59,61,62]; increased angiogenic potential and bone regeneration capacity [51] (compared to bFGF [63])	Short half-life [19,54]
	Clinical trials	Eliminates the pain, scarring, and morbidity of bone harvesting [64,65]; reduces the risk of implant failure; faster healing, fewer infections [66]	Dose-dependent risk of cancer [67]
BMP-7	In vitro	Enhances osteogenic differentiation [68,69,70,71,72]; higher mineralization than in BMP-4 and BMP-4 [69]; lower doses required compared to BMP-2 and BMP-6 [73]; can act as a fibroblast inhibitor [74]	Higher concentration required for osteogenic differentiation, ALP activity, collagen deposition [71,75,76]; cell differentiation rather than proliferation [77]
	In vivo	Improves the healing and the quality of bone tissue [68,78,79]; induces bone formation and tissue calcification [80,81]	Cell differentiation rather than proliferation [73,77]
	Clinical trials	Enhances healing; induces bridging of the bone with an autograft [82]	Dose-dependent risk of cancer [67]
bFGF	In vitro	Induces cell proliferation [69,83,84,85,86,87]; induces osteogenic marker gene expression [88,89,90,91,92,93,94]	Low cytotoxic effect possible [93]; unstable, short half-life [95,96]
	In vivo	Upregulates the expression of osteoblast-related genes [89,92]; promotes bone tissue maturation [85,97,98,99]; upregulates BMP-2 expression [91,94]; enhances osseointegration [88]	Unstable, short half-life [95,96]
	Clinical trials	*	*
Wnt3A	In vitro	Improves cell adhesion and cell density on scaffolds [100]; improves healing [101]; can inhibit osteoclast activity [102]	*
	In vivo	Promotes woven bone formation in critical-size defects [101]	*
	Clinical trials	*	*
IGF-1	In vitro	Improves cell adhesion [103]; induces osteo-differentiation [104,105]	Greater cell adhesion in combination with BMP-2 [103,106]
	In vivo	Improves fracture healing [107,108,109,110]; maintains bone density [111]	Higher healing rate and osteoconductivity in combination with other GFs [103,106,107,108,109,110]
	Clinical trials	Improves wound healing [112]	*
VEGF	In vitro	Enhances cell proliferation [49,113,114,115,116,117,118,119,120,121,122,123]; enhances the effect of BMP-2 [116]; enhances angiogenesis [118,121,124]	*
	In vivo	Improves angiogenic potential and bone regeneration capacity [49,114,116,117,123,124,125,126,127,128,129,130]	Combination with other GFs required for greater effect [98,117,125]
	Clinical trials	*	*
PDGF-BB	In vitro	Induces cell proliferation and enhances osteogenesis [68,69,131]	Increases collagenase activity [132]
	In vivo	Improves healing [133,134,135,136]; induces bone tissue formation [137,138]	Higher bone matrix deposition in combination with other GFs [139]
	Clinical trials	Improves healing of periodontal lesions [112]; maintains crestal bone height [140]	Can have a resorption effect on bone tissue [141]

## Data Availability

The raw/processed data required to reproduce these findings cannot be shared at this time due to legal or ethical reasons.

## Abbreviations

ALP	alkaline phosphatase
bFGF	basic fibroblast growth factor
BHK-21	baby hamster kidney cell
BMP	bone morphogenic protein
BMP-2	bone morphogenic protein-2
BMP-4	bone morphogenic protein-4
BMP-6	bone morphogenic protein-6
BMP-7	bone morphogenic protein-7
BMSC	bone mesenchymal stem cell
COPROG	copolymer-protected gene vector
D-RADA16	biocompatible peptide, comprising arginine (R), alanine (A), and aspartate (D)
ERK1/2	extracellular signal-regulated kinase 1/2
FDA	Food and Drug Administration
FN	fibronectin
GF	growth factor
HA	hydroxyapatite
hAD-MSC	human adipose tissue-derived mesenchymal stem cell
IAPP	ion-assisted plasma polymer
IGF	insulin-like growth factor
IGF-1	insulin-like growth factor-1
miRNA	micro ribonucleic acid
mRNA	messenger ribonucleic acid
MSC	mesenchymal stem cell
NP	nanoparticle
PBMC	peripheral blood mononuclear cell
PCL	poly(ε-caprolactone)
PDLLA	poly(D,L-lactide)
pDNA	plasmid-deoxyribonucleic acid
PEEK	polyetheretherketone
PEG	poly(ethylene glycol)
PLA	polylactic acid
PLGA	poly (lactic-co-glycolic acid)
PRP	platelet-rich plasma
RGD	Arg-Gly-Asp
rhPDGF-BB	recombinant human platelet-derived growth factor BB
Runx2	runt-related transcription factor 2
TGF-β	transforming growth factor-β
TGF-β1	transforming growth factor-β1
TGF-β2	transforming growth factor-β2
Ti-HA	titanium implant infiltrated with hydroxyapatite
VEGF	vascular endothelial growth factor
VEGFA	vascular endothelial growth factor A
Wnt3A	wingless-type MMTV integration site family member 3A
